# The Association Between Neutrophil‐Percentage‐to‐Albumin Ratio (NPAR) and Mortality Among Individuals With Cancer: Insights From National Health and Nutrition Examination Survey

**DOI:** 10.1002/cam4.70527

**Published:** 2025-01-20

**Authors:** Xinyang Li, Meng Wu, Minxin Chen, Rufei Liu, Qingxu Tao, Yun Hu, Jinming Yu, Dawei Chen

**Affiliations:** ^1^ Shandong Provincial Key Laboratory of Precision Oncology, Shandong Cancer Hospital and Institute Shandong First Medical University and Shandong Academy of Medical Sciences Jinan Shandong China; ^2^ Department of Radiation Oncology The University of Texas MD Anderson Cancer Center Houston Texas USA

**Keywords:** cancer, mortality, neutrophil‐percentage‐to‐albumin ratio, survival

## Abstract

**Background:**

Neutrophils interact with tumor cells, potentially exacerbating cancer progression. Additionally, decreased albumin levels are a marker of poor cancer prognosis. The neutrophil‐percentage‐to‐albumin ratio (NPAR) has been used for prognostic assessment in non‐cancerous diseases, but its relationship with mortality risk in cancer patients has not been explored. Therefore, we utilized data from the National Health and Nutrition Examination Survey (NHANES) to investigate the correlation between NPAR and the risks of all‐cause mortality and cancer‐related mortality among cancer patients.

**Methods:**

This study leveraged comprehensive NHANES data spanning 2005–2016. We analyzed the relationship between NPAR and the risks of cancer incidence, all‐cause mortality, and cancer‐related mortality using weighted Logistic and Cox regression models, as well as trend tests. Restricted cubic spline analysis was employed to investigate NPAR's nonlinear relationship with mortality risk. Furthermore, Kaplan–Meier survival analysis was utilized to assess patient prognoses across varying NPAR levels.

**Results:**

Elevated NPAR was associated with an increased risk of all‐cause mortality and cancer‐related mortality in cancer patients (*p* < 0.05), with higher NPAR values correlating with greater risk (*p*‐trend < 0.05). However, no significant association between NPAR and cancer incidence was observed (*p* > 0.05). Our analysis further identified a non‐linear relationship between NPAR and all‐cause mortality risk (*p*‐nonlinear < 0.05), while no non‐linear relationship was found with cancer‐related mortality risk. The relationship is characterized by an optimal NPAR value, correlating with the lowest hazard ratio (HR). Deviations from this optimal NPAR result in increased all‐cause mortality risk (*p* < 0.05). Kaplan–Meier analysis indicated superior survival rates in patients with lower NPAR values compared to those with higher NPAR values (*p* < 0.05).

**Conclusions:**

According to our study, higher NPAR was associated with an increased risk of all‐cause mortality and cancer‐related mortality in cancer patients.

**Abbreviations**
BMI: body mass index
CI: confidence interval
HR: hazard ratio
IQR: interquartile range
NHANES: National Health and Nutrition Examination Survey
NPAR: neutrophil‐percentage‐to‐albumin ratio
OR: odds ratio
PIR: poverty‐to‐income ratio
RCS: restricted cubic spline
ROC: receiver operating characteristic
ROS: reactive oxygen species
WBC: white blood cell

## 1 Introduction

In the mid‐1970s, the 5‐year relative survival rate for cancer was approximately 49%, which improved to 68% between 2012 and 2018 [1]. This notable improvement is largely attributed to advances in early detection and treatment, as well as demographic shifts including population growth and aging [2]. Despite these advancements, cancer continues to be a leading cause of death in the United States and poses a major global public health challenge. The development of enhanced prognostic indicators is imperative for refining treatment strategies and improving survival outcome predictions.

Accurately predicting clinical outcomes in cancer remains challenging due to the complex and not fully understood factors driving cancer progression. Tumor progression often involves extensive vascular neoformation. Neutrophils, which constitute 50%–70% of all circulating leukocytes, are the most abundant myeloid cells in human blood [3]. Despite their prevalence, the implications of their short lifespan, terminally differentiated state, and non‐proliferative nature on their functional significance in cancer progression are often underappreciated. The link between inflammation and cancer progression is well‐established, with numerous studies highlighting the role of inflammatory cells, [4] growth factors, [5] activated stroma [6], and DNA‐damaging agents [7] in tumor progression. As primary responders to acute inflammation, neutrophils play a pivotal role in tumor progression [8]. Substantial evidence supports the role of neutrophils in cancer promotion. The pro‐carcinogenic effect of neutrophils, primarily through the release of reactive oxygen species (ROS), results in oxidative DNA damage in the lungs, [9] and an increased mutation burden in inflammation‐driven colorectal cancer models [10, 11]. Moreover, a dynamic interplay between cancer cells and neutrophils has been observed in the tumor microenvironment [12]. Neutrophils are characterized by their high plasticity, [13, 14] significantly contributing to their functional diversity in cancer progression. A critical aspect of neutrophils' role in cancer is their capacity to suppress the anti‐cancer activities of other immune cells [15]. These mechanisms collectively underscore the role of neutrophils in promoting cancer. Albumin, constituting 50% of plasma proteins in healthy individuals, is crucial for fluid balance, nutrient and drug transport, and immune protection [16]. In cancer patients, albumin levels often decrease due to factors like the high metabolic demands of cancer cells and nutrient competition. Low albumin concentrations are associated with increased mortality, [17] and advanced non‐small cell lung cancer patients often exhibit malnutrition, characterized by subnormal albumin levels [18].

The neutrophil‐percentage‐to‐albumin ratio (NPAR) serves as a cost‐effective and accessible biomarker for inflammation, aiding in prognostication for conditions like advanced liver fibrosis, [19] acute kidney injury, [20] and cardiovascular diseases [21, 22]. Both neutrophils and albumin play crucial roles in inflammation and immune responses, with emerging evidence linking these responses to the efficacy of cancer therapies and patient prognoses [23, 24, 25, 26, 27]. Despite this, there is a notable gap in research regarding the relationship between NPAR and cancer mortality, and its potential as a prognostic tool. Therefore, this study aims to explore the possible association of NPAR with cancer‐related mortality, hypothesizing that NPAR could be a critical indicator of cancer prognosis.

## 2 Methods

### 2.1 Study Population

**FIGURE 1:**
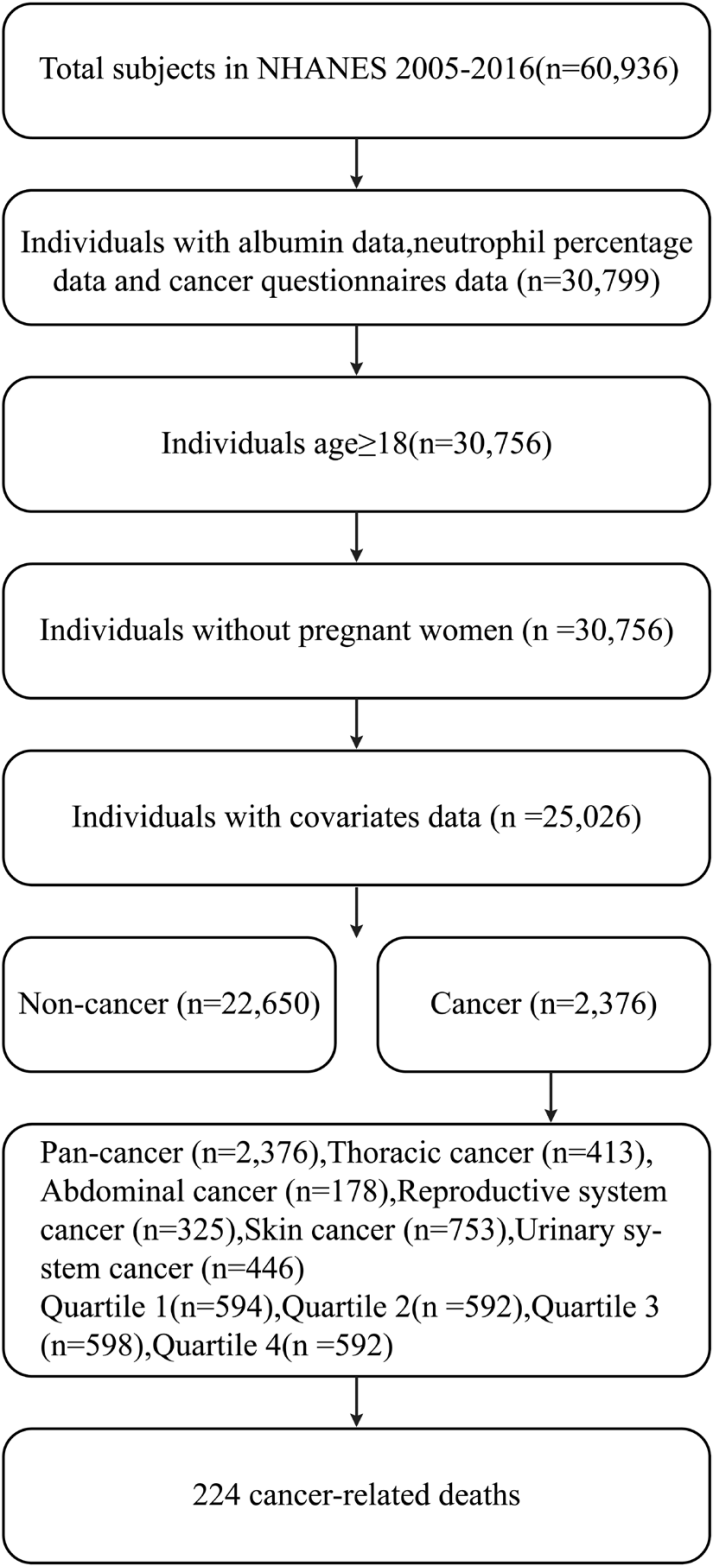
Flowchart illustrating selection criteria of the study population. Population selection (*n* = 25,026) for analysis of the association between NPAR and cancer. And the population selection of various cancers (*n* = 2374) was presented to analyze the relationship between NPAR and cancer mortality. The 10th revised edition (ICD‐10) based on the International Statistical Classification of Diseases and Related Health Problems defined the cause of cancer mortality: malignant neoplasms (019‐043).

The study utilized data from the NHANES database, which assesses the health and nutritional status of the U.S. adult and child population through interviews and physical examinations. Since 1999, the survey has been conducted biennially, providing a continuous source of information on the health and nutritional status of the American population (NHANES. https://www.cdc.gov/nchs/nhanes/about_nhanes). The study protocol was approved by the National Center for Health Statistics and the Research Ethics Review Committee of the Centers for Disease Control and Prevention. And written informed consent was provided by all participants or agents who were selected through a complex, multi‐stage probability sampling design. The study utilized NHANES data from 2005 to 2016, focusing on participants aged over 18 years. Exclusions were made for participants with incomplete cancer questionnaires, missing neutrophil and serum albumin data, pregnancy, or lacking other covariates data. The final analysis included 25,026 participants, comprising 22,650 non‐cancer cases and 2376 cancer patients (Figure 1).

### 2.2 Covariates Collection and Definitions

Demographic data, including age, sex, ethnicity, marital, poverty‐to‐income ratio (PIR), smoking status, alcohol assumption status, and educational level, were collected by trained NHANES interviewers through in‐person interviews using the Family and Sample Person Demographics questionnaires and the Computer‐Assisted Personal Interviewing (CAPI) system (Confirmit Corp., New York, NY, USA).

Hematologic parameters were measured using the NHANES CBC Profile with the Beckman Coulter Automated Hematology Analyzer DxH 900 (Beckman‐Coulter, Brea, CA, USA). This system evaluates red and white cell counts, hemoglobin, hematocrit, and red blood cell indices. The Coulter VCS system was employed for white blood cell (WBC) differential analysis. The analyzer employs an automated dilution and mixing system for cell counting and sizing, along with a single‐beam photometer for hemoglobin measurement. Lymphocyte counts were measured using the same method as for neutrophil counts. The NPAR was determined for each participant using the following formula: [Neutrophil percentage (of total WBC count)/Albumin (g/dL)] × 100.

Body mass index (BMI) was calculated as body weight (in kilograms) divided by height (in meters squared). Hypertension was defined as a self‐reported or physician's diagnosed condition, with systolic blood pressure or diastolic blood pressure meeting certain thresholds such as ≥ 140 or ≥ 90 mmHg, or using medication for blood pressure control [28]. Diabetes was identified by HbA1c ≥ 6.5%, serum glucose at 2 h following a 75‐g glucose load (OGTT) ≥ 200 mg/dL, fasting glucose ≥ 126 mg/dL, self‐reported diagnosis of diabetes, or use of insulin or other diabetes medication [29]. Hyperlipidemia was defined as higher‐than‐normal plasma lipid levels [30].

In 2022, the National Center for Health Statistics in the United States released the NHANES 1999–2018 Linked Mortality File (LMF), encompassing mortality status and causes of death. International Classification of Diseases (ICD‐10) was used to determine disease‐specific deaths. Cancer was diagnosed based on information on comorbidities from the household interview questionnaire, encompassing all types of cancer recorded in NHANES [31]. Cancer‐related mortality was defined as codes C00‐C97 [32]. Participants lacking reliable identification data or those with non‐disclosable data were excluded from mortality analysis in this study.

### 2.3 Statistical Analysis

All data analyses in this study were conducted in accordance with NHANES guidelines. Descriptive statistics were applied to demographic and anthropometric data, with sample weights derived from NHANES' complex sample design. Continuous variables were analyzed using means and standard errors, and categorical variables were assessed using weighted counts and proportions. *t*‐Tests and chi‐square tests were applied for comparison of continuous variables and categorical variables, respectively. NPAR was analyzed as both a continuous and categorical variable (divided into quartiles). The relationship between NPAR and cancer incidence was evaluated using weighted logistic regression, with cancer as the dependent variable and NPAR as the independent variable. Multivariable Cox regression analysis was employed to ascertain the association between NPAR and all‐cause mortality and cancer‐related mortality additionally adjusted for age, gender, ethnicity, education level, marital, drinking status, smoking status, PIR, hypertension, hyperlipidemia, and diabetes. Similarly, multivariable Cox regression analysis was employed to ascertain the association between NPAR and all‐cause mortality among prostate cancer patients in NHANES 2005–2010 (only the prostate cancer sample data from 2005 to 2010 includes treatment method information) adjusted for age, gender, ethnicity, education level, marital, drinking status, smoking status, PIR, surgery, radiotherapy, medicine, hypertension, hyperlipidemia, and diabetes. Kaplan–Meier survival curves were used for comparing survival probabilities. Restricted cubic spline (RCS) analysis was used to investigate the relationship between NPAR and various cancer types. Propensity score matching was used to find balanced and matched subjects. The receiver operating characteristic (ROC) curve was used for reporting an optimal cut‐off with related characteristics (sensitivity, specificity, and area under the curve). A post hoc test was used to explore variables with significant distributions between the quartiles of NPAR. Statistical significance was defined as a two‐sided *p* value less than 0.05. Statistical analyses were conducted using R software (version 3.6.3, http://www.R‐project.org/).

## 3 Results

### 3.1 Study Participants and Baseline Characteristics

The baseline characteristics are summarized in Table 1. The average age was 47.46 years (50.29% male, 49.71% female). The cancer patients had a median age of 62.46 years, significantly older than the non‐cancer participants (45.81 years). Higher cancer incidence was noted among non‐Hispanic whites, those with an education level of high school or above, married or cohabiting individuals, alcohol consumers, PIR > 1, and those with hypertension, hyperlipidemia, low albumin levels, and higher NPAR. The average BMI was 28.95 kg/m^2^. Notably, in the non‐cancer group, albumin levels were higher, and neutrophil proportion, as well as NPAR were lower. We conducted propensity score matching on the aforementioned data and, after verifying the initial equilibrium and assessing the quality of the match, we successfully matched 2376 cancer patients with 2376 non‐cancer participants. Analysis of the matched data (Table S1) revealed that compared to non‐cancer participants, the cancer group exhibited higher levels of NPAR with statistical significance (*p* = 0.014); however, variables such as age, gender, ethnicity, education, marital status, drinking, smoking, BMI, PIR, hyperlipidemia, albumin, neutrophil percent, neutrophil count, and lymphocyte count showed no statistically significant differences between the two groups after eliminating sample size discrepancies.

**TABLE 1:** Baseline characteristics of study participants in NHANES 2005–2016.

Variables	NPAR	Total participant, *N* = 25,026	Non‐cancer, *N* = 22,650	Cancer, *N* = 2376	*p*
Age, years	—	47.46 (0.26)	45.81 (0.26)	62.46 (0.39)	< 0.0001
Gender	13.69				< 0.0001
Male	13.38	12,586 (50.29)	11,457 (50.19)	1129 (43.44)	
Female	14.00	12,440 (49.71)	11,193 (49.81)	1247 (56.56)	
Ethnicity	13.69				< 0.0001
Non‐Hispanic white	13.83	11,521 (46.04)	9824 (68.56)	1697 (87.82)	
Non‐Hispanic black	13.00	5041 (20.14)	4728 (10.76)	313 (4.69)	
Mexican American	13.68	3888 (15.54)	3744 (8.57)	144 (2.12)	
Other race	13.45	4576 (18.28)	4354 (12.11)	222 (5.37)	
Education	13.69				< 0.0001
Below high school level	13.85	6074 (24.27)	5584 (16.21)	490 (12.38)	
High school	13.80	5743 (22.95)	5213 (22.77)	530 (20.70)	
Above high school	13.61	13,209 (52.78)	11,853 (61.02)	1356 (66.92)	
Marital	13.69				< 0.0001
Married/living with partner	13.65	15,027 (60.05)	13,581 (64.03)	1446 (65.68)	
Widowed/divorced/separated	14.20	5591 (22.34)	4805 (17.45)	786 (28.52)	
Never married	13.31	4408 (17.61)	4264 (18.52)	144 (5.80)	
Drinking	13.69				< 0.0001
Never	13.79	3492 (13.95)	3186 (10.93)	306 (10.21)	
Former	14.12	4600 (18.38)	4014 (14.73)	586 (19.92)	
Current	13.59	16,934 (67.67)	15,450 (74.34)	1484 (69.86)	
Smoking	13.69				< 0.0001
Never	13.56	13,498 (53.94)	12,441 (55.02)	1057 (45.42)	
Former	13.82	6240 (24.93)	5296 (23.74)	944 (38.46)	
Current	13.87	5288 (21.13)	4913 (21.24)	375 (16.12)	
BMI (kg/m^2^)	—	28.95 (0.08)	28.96 (0.09)	28.87 (0.15)	0.57
Poverty‐to‐income ratio	13.69				< 0.0001
Poor (≤ 1)	13.76	5245 (20.96)	4897 (14.36)	348 (9.25)	
Not poor (> 1)	13.68	19,781 (79.04)	17,753 (85.64)	2028 (90.75)	
Hypertension	13.69				< 0.0001
No	13.48	14,387 (57.49)	13,531 (64.44)	856 (42.44)	
Yes	14.05	10,639 (42.51)	9119 (35.56)	1520 (57.56)	
Hyperlipidemia	13.69				< 0.0001
No	13.49	7007 (28)	6601 (30.01)	406 (16.97)	
Yes	13.77	18,019 (72)	16,049 (69.99)	1970 (83.03)	
Diabetes	13.69				< 0.0001
No	13.56	20,392 (81.48)	18,662 (87.01)	1730 (78.09)	
Yes	14.52	4634 (18.52)	3988 (12.99)	646 (21.91)	
NPAR	—	13.69 (0.03)	13.64 (0.03)	14.21 (0.07)	< 0.0001
Q1	—	6250 (24.97)	5821 (24.73)	429 (18.46)	
Q2	—	6256 (25)	5759 (26.65)	497 (22.85)	
Q3	—	6262 (25.02)	5632 (25.38)	630 (27.78)	
Q4	—	6258 (25.01)	5438 (23.24)	820 (30.91)	
Albumin, g/dL	—	4.30 (0.00)	4.30 (0.00)	4.23 (0.01)	< 0.0001
Neutrophil percent, %	—	58.44 (0.11)	58.30 (0.11)	59.71 (0.25)	< 0.0001
Neutrophil count, 10^9^/L	—	4.30 (0.02)	4.30 (0.02)	4.32 (0.05)	0.65
Lymphocyte count, 10^9^/L	—	2.13 (0.01)	2.14 (0.01)	2.07 (0.03)	0.04

*Note:* The continuous variables were analyzed by *t*‐test, expressed by the median (IQR); the weighted chi‐square test was used to analyze the categorical variables, expressed by the column percentage. A level of two‐sided *p* < 0.05 was considered statistically significant. Means and standard error were described for the continuous variables, counts, and proportions (after weighted) were described for categorical variables.

Abbreviations: BMI, body mass index; IQR, interquartile range; NHANES, National Health and Nutrition Examination Survey; NPAR, neutrophil‐percentage‐to‐albumin ratio.

### 3.2 Association of NPAR With Cancer Risk: NHANES 2005–2016

The association between NPAR and cancer risk was analyzed using weighted multifactor Logistic regression, both as a categorical and continuous variable. The findings are detailed in Table S2. Initial univariate logistic regression revealed an increased likelihood of cancer in higher NPAR quartiles: the odds increased by 47% (OR = 1.47 [95% CI 1.20, 1.79]) for the third quartile (Q3) and 78% (OR = 1.78 [95% CI 1.49, 2.13]) for the fourth quartile (Q4), compared to the first quartile (Q1). A continuous variable analysis of NPAR also demonstrated a positive correlation with cancer risk (OR = 1.10 [95% CI 1.07, 1.12]). Subsequent multivariable logistic regression, adjusted for factors like age, sex, race, smoking, alcohol consumption, PIR, hypertension, hyperlipidemia, and diabetes (Table S2), yielded odds ratios for Q2, Q3, and Q4 of 0.97 (95% CI 0.76, 1.25), 0.99 (95% CI 0.69, 1.41), and 0.98 (95% CI 0.60, 1.59), respectively. Continuous analysis under this model showed similar findings (OR = 1.07 [95% CI 0.84, 1.36]). Notably, *p*‐values in both categorical and continuous analyses were above 0.05, suggesting no statistically significant association.

### 3.3 Association of NPAR With the Risk of All‐Cause Mortality and Cancer‐Related Mortality Among Cancer Patients: NHANES 2005–2016

**FIGURE 2:**
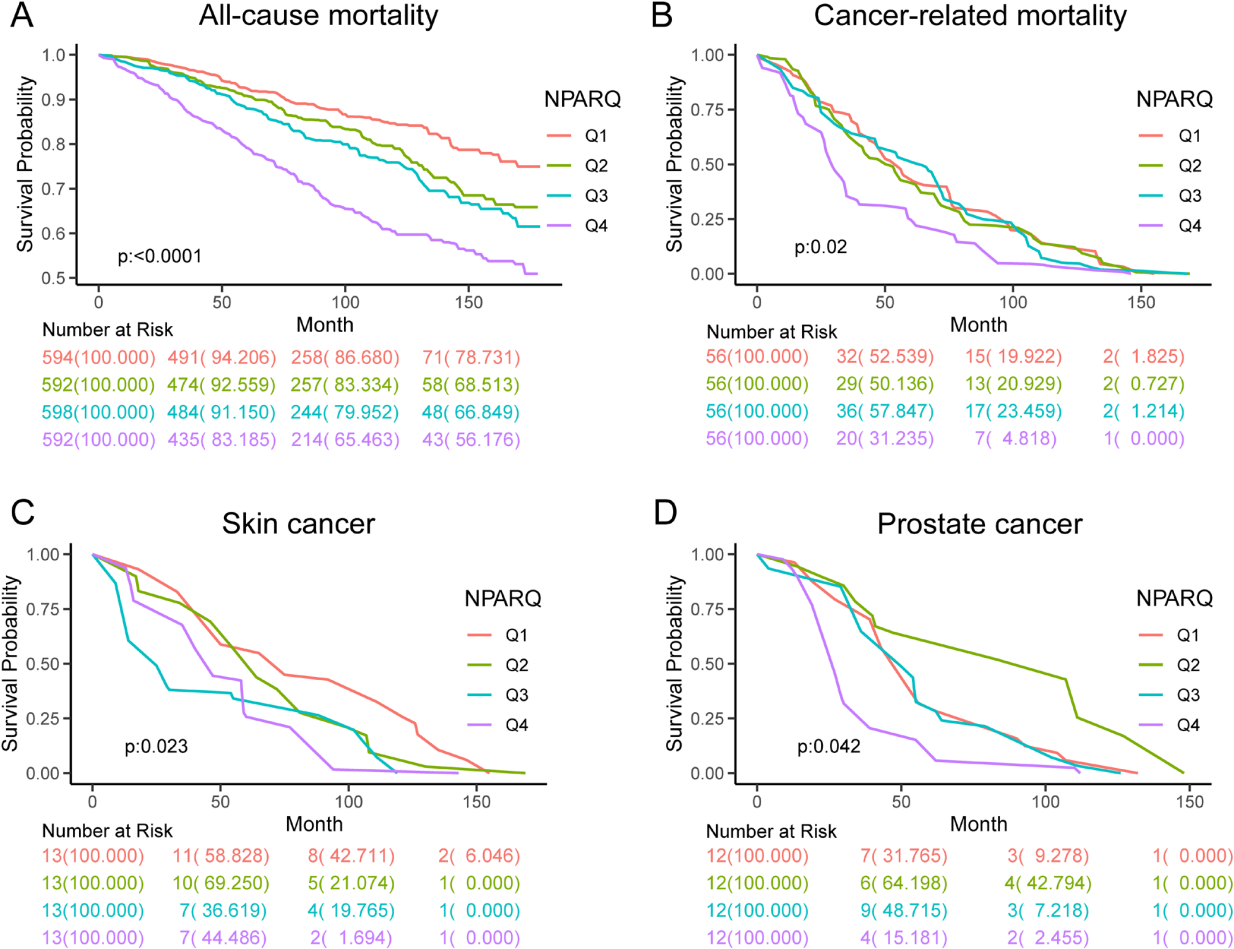
Kaplan–Meier survival curves for mortality outcomes. (A) All‐cause mortality, (B) cancer‐related mortality, (C) skin cancer‐related mortality, and (D) prostate cancer‐related mortality.

Follow‐up duration was calculated in person‐months from the interview date to either the date of death or the end of the follow‐up period. During the 11‐year follow‐up, 2376 cancer patients were recorded, including 224 cancer‐related deaths. Basic demographic and clinical data of cancer patients stratified by NPAR quartiles are detailed in Table S3. Cancer types were classified into categories: thoracic (breast, lung, and esophageal), abdominal (colorectal, stomach, liver, gallbladder, and pancreatic), reproductive system (uterine, cervical, ovarian, and testicular), urinary system (kidney, prostate, and bladder), and skin (melanoma and other skin tumors). Adjusted analyses revealed a positive correlation between NPAR and all‐cause mortality as well as cancer‐related mortality. The relationship was examined in both continuous and categorical formats, assessing all‐cause mortality risks across various cancer types: overall, thoracic, abdominal, reproductive system, skin, and urinary system tumors (Table 2). In the analysis of overall all‐cause mortality, continuous NPAR showed an adjusted hazard ratio (aHR) of 1.08 (95% CI 1.04, 1.12); while categorical analysis indicated an increased risk in Q4 compared to Q1 with an aHR of 1.73 [95% CI 1.34, 2.22], both significant (*p* < 0.05). Thoracic tumor analysis revealed an aHR of 1.07 (95% CI 0.99, 1.16) for continuous NPAR and no significant differences in categorical among Q2 (aHR 1.18, 95% CI 0.60, 2.29), Q3 (aHR 1.07, 95% CI 0.59, 1.96), and Q4 (aHR 1.50, 95% CI 0.80, 2.82) against Q1. For abdominal tumors, continuous NPAR analysis showed an aHR of 1.14 (95% CI 1.04, 1.26). Categorically, Q4 showed a significantly higher risk than Q1 (aHR = 2.61 [95% CI 1.30, 5.24]), both *p* < 0.05. In reproductive system tumors, aHR were 1.07 (95% CI 0.90, 1.27) and 0.89 (95% CI 0.24, 3.32), with no significant differences. Skin tumor analysis showed a 14% increased risk for continuous NPAR (aHR = 1.14 [95% CI 1.05, 1.24]) and a 70% increase for Q4 compared to Q1 in categorical analysis (aHR = 1.70 [95% CI 1.04, 2.77]), both statistically significant. Urinary system tumors analysis found an aHR of 1.07 (95% CI 0.97, 1.17) for continuous NPAR and an increased, but not significant, risk for Q4 compared to Q1 (aHR = 1.53 [95% CI 0.88, 2.63]). Trend tests were also conducted for various tumor types. A significant upward trend in all‐cause mortality risk was observed with increasing NPAR levels in overall cancer, abdominal, skin, and urinary system tumors, with respective *p*‐values for the trend as follows: < 0.0001, < 0.005, < 0.02, and < 0.048 (Table 2). Figure 2A demonstrates a statistically significant difference in survival probability among four NPAR groups in mortality outcomes.

**TABLE 2:** The relationship between NPAR and all‐cause mortality among cancer patients in NHANES 2005–2016.

Cancer	Character	Range median (IQR)	aHR^a^ (95% CI)	*p*
Pan‐cancer	NPAR		1.08 (1.04, 1.12)	< 0.0001
	Q1	11.43 (0.86–12.67)	Ref	Ref
	Q2	13.55 (12.67–14.31)	1.22 (0.92, 1.62)	0.16
	Q3	15.12 (14.31–16.05)	1.16 (0.89, 1.51)	0.28
	Q4	17.41 (16.05–36.10)	1.73 (1.34, 2.22)	< 0.0001
	*p* for trend			< 0.0001
Thoracic cancer	NPAR		1.07 (0.99, 1.16)	0.08
	Q1	11.468 (6.95–12.93)	Ref	Ref
	Q2	13.884 (12.93–14.70)	1.18 (0.60, 2.29)	0.63
	Q3	15.366 (14.70–16.56)	1.07 (0.59, 1.96)	0.82
	Q4	18.244 (16.56–29.47)	1.50 (0.80, 2.82)	0.21
	*p* for trend			0.232
Abdominal cancer	NPAR		1.14 (1.04, 1.26)	0.004
	Q1	11.67 (4.16–12.91)	Ref	Ref
	Q2	13.89 (12.91–14.39)	1.09 (0.37, 3.18)	0.88
	Q3	15.05 (14.39–16.11)	1.54 (0.74, 3.21)	0.25
	Q4	17.60 (16.11–28.07)	2.61 (1.30, 5.24)	0.01
	*p* for trend			0.005
Reproductive system cancer	NPAR		1.07 (0.90, 1.27)	0.46
	Q1	11.45 (8.28–12.67)	Ref	Ref
	Q2	13.52 (12.67–13.98)	1.10 (0.30, 4.04)	0.88
	Q3	14.84 (13.98–15.73)	0.62 (0.11, 3.57)	0.6
	Q4	16.77 (15.73–21.79)	0.89 (0.24, 3.32)	0.87
	*p* for trend			0.718
Skin cancer	NPAR		1.14 (1.05, 1.24)	0.002
	Q1	11.55 (2.62–12.51)	Ref	Ref
	Q2	13.39 (12.51–14.16)	1.25 (0.71, 2.23)	0.44
	Q3	15.05 (14.16–15.91)	1.52 (0.91, 2.53)	0.11
	Q4	16.93 (15.91–36.10)	1.70 (1.04, 2.77)	0.03
	*p* for trend			0.02
Urinary system cancer	NPAR		1.07 (0.97,1.17)	0.18
	Q1	11.52 (1.35–12.75)	Ref	Ref
	Q2	13.68 (12.75–14.46)	0.90 (0.53, 1.55)	0.71
	Q3	15.13 (14.46–16.24)	1.10 (0.64, 1.88)	0.73
	Q4	18.07 (16.24–22.35)	1.53 (0.88, 2.63)	0.13
	*p* for trend			0.048

*Note:* Cox proportional hazards regression for the relationship between NPAR and mortality among cancer patients.

Abbreviations: CI, confidence interval; HR, hazard ratio; IQR, interquartile range; NPAR, neutrophil percentage‐to‐albumin ratio.

^a^ Adjusted for age, gender, ethnicity, education level, marital, drinking status, smoking status, poverty‐to‐income ratio, hypertension, hyperlipidemia, and diabetes.

In our subsequent analysis of the relationship between NPAR and cancer‐related mortality, we found that continuous NPAR was associated with an aHR of 1.06 (95% CI 1.01, 1.12; *p* = 0.03). Categorical analysis further revealed a heightened risk in Q4 compared to Q1, with an aHR of 1.99 (95% CI 1.29, 3.08; *p* = 0.002). The trend in pan‐cancer mortality demonstrated a statistically significant *p*‐value of < 0.001 (Table 3). Moreover, the Kaplan–Meier survival analysis demonstrated significant disparities in survival probabilities among the NPAR quartiles (Q1, Q2, Q3, and Q4) within a cohort of 224 patients, as depicted in Figure 2B. Furthermore, subgroup analyses revealed notable distinctions in survival probabilities across these quartiles specifically in patients diagnosed with skin cancer and prostate cancer (Figure 2C,D). However, in breast cancer, we observed no apparent disparities (Figure S1).

**TABLE 3:** The relationship between NPAR and cancer‐related mortality among cancer patients in NHANES 2005–2016.

Cancer	Character	Range median (IQR)	aHR^a^ (95% CI)	*p*
Pan‐cancer (*n* = 224)	NPAR		1.06 (1.01, 1.12)	0.03
	Q1	11.35 (0.86–12.78)	Ref	Ref
	Q2	13.72 (12.78–14.66)	1.08 (0.72, 1.62)	0.72
	Q3	15.54 (14.66–16.68)	1.31 (0.84, 2.03)	0.23
	Q4	18.27 (16.68–29.47)	1.99 (1.29, 3.08)	0.002
	*p* for trend			< 0.001

*Note:* Cox proportional hazards regression for the relationship between NPAR and mortality among cancer patients.

Abbreviations: CI, confidence interval; HR, hazard ratio; IQR, interquartile range; NPAR, neutrophil percentage‐to‐albumin ratio.

^a^ Adjusted for age, gender, ethnicity, education level, marital, drinking status, smoking status, poverty‐to‐income ratio, hypertension, hyperlipidemia, and diabetes.

Considering the contributing factors to cancer mortality are mainly the initial cancer stage, antineoplastic treatment modalities such as surgery, radiotherapy, and access to antineoplastic drugs, we analyzed data from prostate cancer questionnaires conducted between 2005 and 2010 (Table S4). The reason is that only the survey questionnaire for prostate cancer includes information on treatment. By adjusting for confounding variables including surgical, radiotherapeutic, and pharmacological treatments, our results revealed an aHR of 1.22 (95% CI 1.03, 1.44), *p* = 0.02. Categorically, Q2 and Q4 showed a significantly higher risk than Q1 (aHR = 6.07 [95% CI 1.52, 24.24], aHR = 8.71 [95% CI 1.43, 53.24]), both *p* < 0.05 (Table S5).

### 3.4 RCS Analysis

**FIGURE 3:**
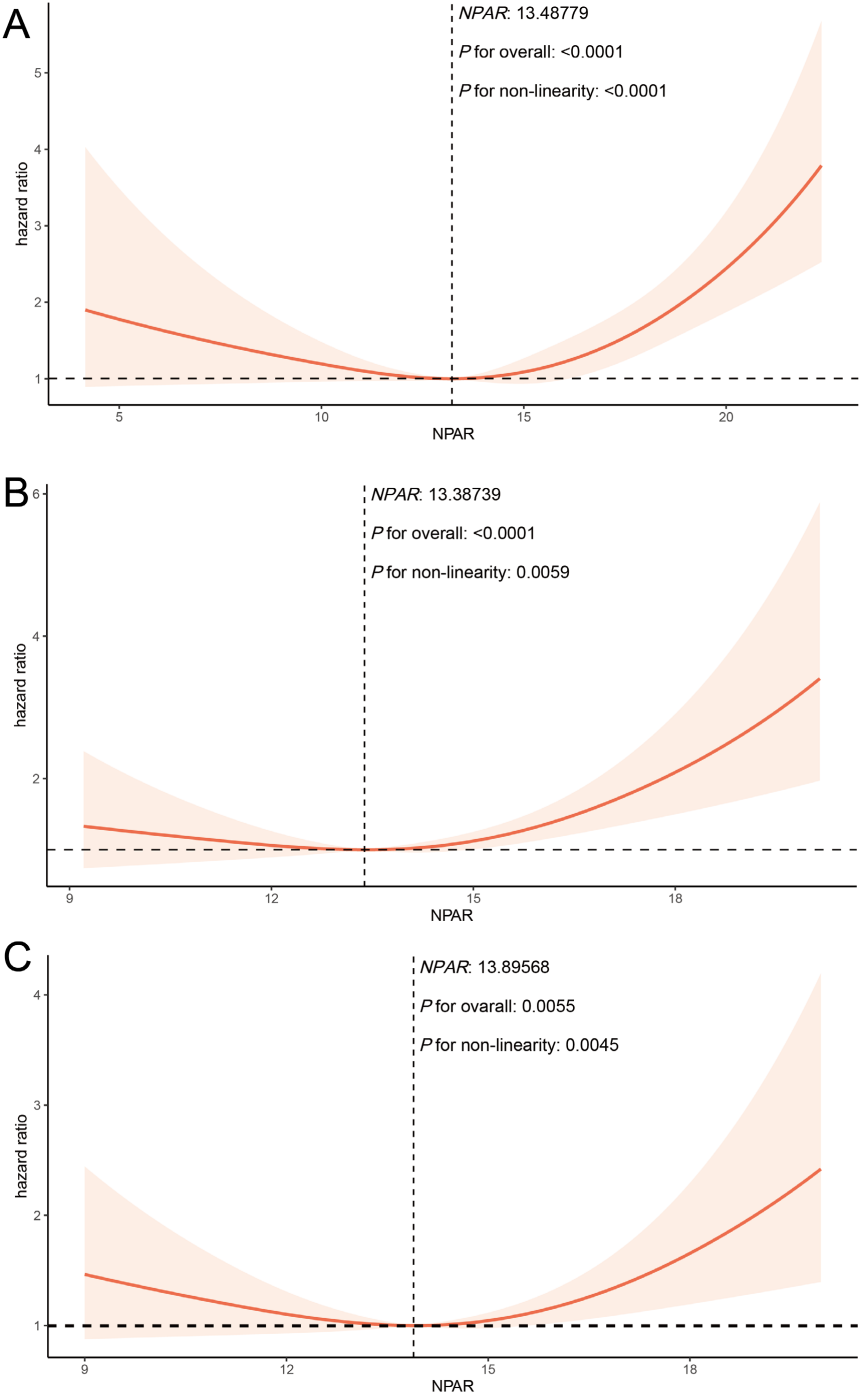
Restricted cubic spline analysis of the nonlinear relationship between continuous NPAR and hazard ratio. Non‐linear relationship of NPAR and the risk of all‐cause mortality in pan‐cancer (A), skin cancer (B), and urinary system cancer (C).

Beyond linear analysis, the study also investigated the potential non‐linear relationship between NPAR and all‐cause mortality (Figure 3) and cancer‐related mortality (Figure S1). Utilizing RCS analysis for model adjustment revealed a significant non‐linear relationship between NPAR and overall all‐cause mortality (*p* < 0.0001) (Figure 3A). Specifically, all‐cause mortality risk decreased to its lowest at an NPAR level of 13.48779, then increased as NPAR rose beyond this point (*p* < 0.0001). This non‐linear trend was also evident in skin cancer and urinary system tumors (*p* < 0.05) (Figure 3B,C). For NPAR levels below 13.38739 and 13.89568, all‐cause mortality risk declined with rising NPAR; on the contrary, exceeding these thresholds led to increased mortality risk. This non‐linear pattern was not observed in the other tumor types. Moreover, there was no nonlinear relationship between cancer‐related mortality and NPAR (Figure S2).

### 3.5 Post‐Hoc Tests

To verify the reliability of the research results and explore which quartile of NPAR differs from the others as well as the differences in patient prognosis among different quartile groups, we conducted post‐hoc tests on the data of all cancer‐related death patients. We found significant statistical differences between all quartiles of NPAR (Table S6). In addition, compared to the other three groups, patients in the Q4 group had the worst prognosis, with no significant statistical difference among the remaining three groups (Table S7).

### 3.6 Sensitivity Analysis

There is a significant association between NPAR and all‐cause mortality as well as cancer‐related mortality. We aim to find the optimal cut‐off to reflect the relationship between NPAR and mortality. Based on the NPAR values and survival status of 2376 tumor patients, the receiver operating characteristic (ROC) curve was constructed (Figure S3). The optimal cutoff value was 14.21429, with an AUC of 0.61, sensitivity of 0.6412104, and specificity of 0.5311377. Due to all the survival information of patients who died from cancer being in a deceased state, we did not perform an ROC curve analysis on them.

## 4 Discussion

Research on the role of NPAR in cancer is relatively scarce, highlighting the innovativeness of this study. This study established a connection between NPAR levels in cancer patients and the risks of all‐cause mortality and cancer‐related mortality using the NHANES database. For all‐cause mortality risk in cancer patients, Kaplan–Meier survival analysis and Cox proportional hazards models indicated that elevated NPAR levels were associated with a higher risk of all‐cause mortality, including for pan‐cancer, abdominal tumors, skin, and urological cancers. Trend analysis confirmed a positive correlation between NPAR and all‐cause mortality risk, suggesting that each unit increase in NPAR was related to a higher all‐cause mortality rate among cancer patients. However, RCS regression indicated a subtle nonlinear relationship between NPAR and all‐cause mortality risk for pan‐cancer, skin, and urological cancers. Specifically, when NPAR levels were between 13 and 14, all‐cause mortality risk decreased, while levels exceeding this range were associated with increased risk, indicating that both excessively low and high NPAR levels could elevate the risk of all‐cause mortality. Notably, the RCS analysis primarily illustrated the changes in aHR corresponding to each unit change in NPAR as a continuous variable, while the remaining aHR values were derived from the aHR value at the cut point (aHR = 1). Figure 3A showed that, overall, higher NPAR was associated with a greater risk of all‐cause mortality, indirectly validating the results of the p for trend test. On the other hand, we found a positive correlation between NPAR in the overall cancer population and cancer‐related mortality risk. Kaplan–Meier survival analysis revealed that patients with higher NPAR levels in pan‐cancer, breast cancer, and skin cancer had poorer prognoses.

Neutrophils significantly influence cancer progression by protecting circulating tumor cells, thereby enhancing dissemination and metastasis [33, 34]. The detection of tumor cell‐neutrophil clusters in the bloodstream correlates with a worse prognosis in breast cancer patients [35]. Albumin, a vital medium‐sized carrier protein, is involved in various functions including osmotic regulation, antioxidation, anti‐inflammatory effects, nutrient and drug transport, and acid–base balance regulation, constituting over half of the total serum proteins. Conditions like liver cancer and cirrhosis not only reduce albumin synthesis but also alter its structure and function [36, 37, 38]. NPAR's prognostic value has been documented in pancreatic cancer, [39] septic shock, [40] atrial fibrillation, [41] and liver cirrhosis [42]. The elevated levels of neutrophils and higher tumor burden during tumor progression lead to a decrease in the body's own albumin levels, which may explain why increased NPAR levels are associated with a higher risk of mortality.

This study has several limitations. First, it is an observational study, and therefore residual confounding factors cannot be ruled out. However, many covariates have been adjusted, and various statistical testing methods have been employed to minimize confounding. Second, the diagnosis of cancer is based on self‐reported data from questionnaires, without standardized medical records, which may be subject to recall bias. Nevertheless, all interviewers were well‐trained and utilized a CAPI to reduce data entry errors. Third, the number of cancer patients with specific cancer‐related deaths, such as those from lung cancer, esophageal cancer, and colorectal cancer, is minimal, hindering our ability to further explore the relationship between NPAR and cancer‐related mortality. Fourth, due to the limited number of deaths, detailed cancer information is lacking. Therefore, more large‐scale studies, including clinical trials, are needed.

In summary, elevated NPAR is associated with an increased risk of all‐cause mortality and cancer‐related mortality in cancer patients.

## Author Contributions

**Xinyang Li:** conceptualization (lead), data curation (lead), formal analysis (lead), investigation (lead), methodology (lead), visualization (lead), writing – original draft (lead), writing – review and editing (lead). **Meng Wu:** funding acquisition (supporting), investigation (supporting), project administration (supporting), writing – review and editing (supporting). **Minxin Chen:** investigation (supporting). **Rufei Liu:** investigation (supporting). **Qingxu Tao:** investigation (supporting). **Yun Hu:** writing – review and editing (supporting). **Jinming Yu:** conceptualization (supporting), funding acquisition (supporting), project administration (supporting), supervision (supporting), writing – review and editing (supporting). **Dawei Chen:** conceptualization (supporting), funding acquisition (supporting), methodology (supporting), project administration (supporting), supervision (supporting), writing – review and editing (supporting).

## Ethics Statement

The manuscript of the data from the NHANES database (https://www.cdc.gov/nchs/nhanes/about_nhanes.htm), and the National Center for Health Statistics and the Research Ethics Review Committee of the Centers for Disease Control and Prevention approved the investigation plan.

## Conflicts of Interest

The authors declare no conflicts of interest.

## Supporting information


Figures S1–S3.

Table S1.

Table S2.

Table S3.

Table S4.

Table S5.

Table S6.

Table S7.

## Data Availability

We confirm that we have included a citation for available data in our references section.
