# Supplementary material for: The Association Between Neutrophil‐Percentage‐to‐Albumin Ratio (NPAR) and Mortality Among Individuals With Cancer: Insights From National Health and Nutrition Examination Survey
Source: Cancer Med. 2025 Jan 20;14(2):e70527. doi: 10.1002/cam4.70527 (PMC11744675; doi:10.1002/cam4.70527)
Supplement: Supplementary file 1 — Figures S1–S3. [file CAM4-14-e70527-s008.zip › cam470527-sup-0011-Supinfo.docx]

Figure S1. Kaplan-Meier survival curves for breast cancer mortality outcomes.

Figure S2. Non-linear relationship of NPAR and the risk of cancer-related mortality in pan-cancer.

Figure S3. Receiver Operating Characteristic (ROC) curve based on the NPAR values and survival status of 2376 tumor patients.
