# Supplementary figures and images for: The Association Between Neutrophil‐Percentage‐to‐Albumin Ratio (NPAR) and Mortality Among Individuals With Cancer: Insights From National Health and Nutrition Examination Survey
Source: Cancer Med. 2025 Jan 20;14(2):e70527. doi: 10.1002/cam4.70527 (PMC11744675; doi:10.1002/cam4.70527)

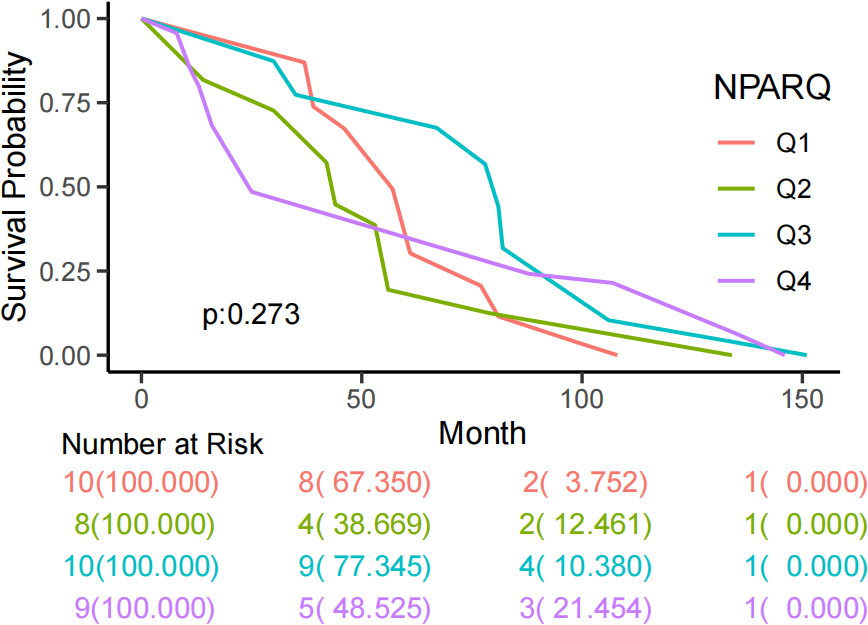

Supplement: Supplementary file 1 — Figures S1–S3. [file CAM4-14-e70527-s008.zip › cam470527-sup-0001-FigureS1.tif]

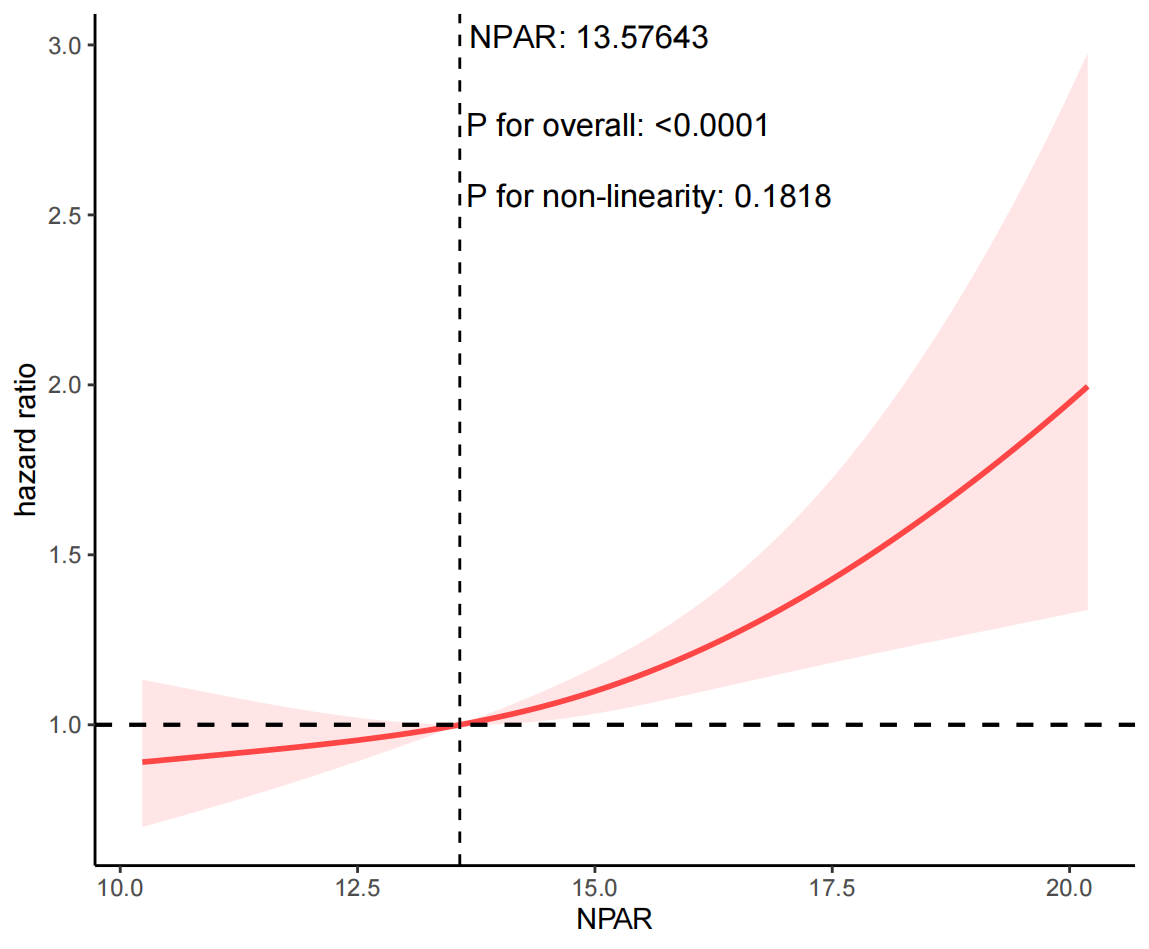

Supplement: Supplementary file 1 — Figures S1–S3. [file CAM4-14-e70527-s008.zip › cam470527-sup-0002-FigureS2.tif]

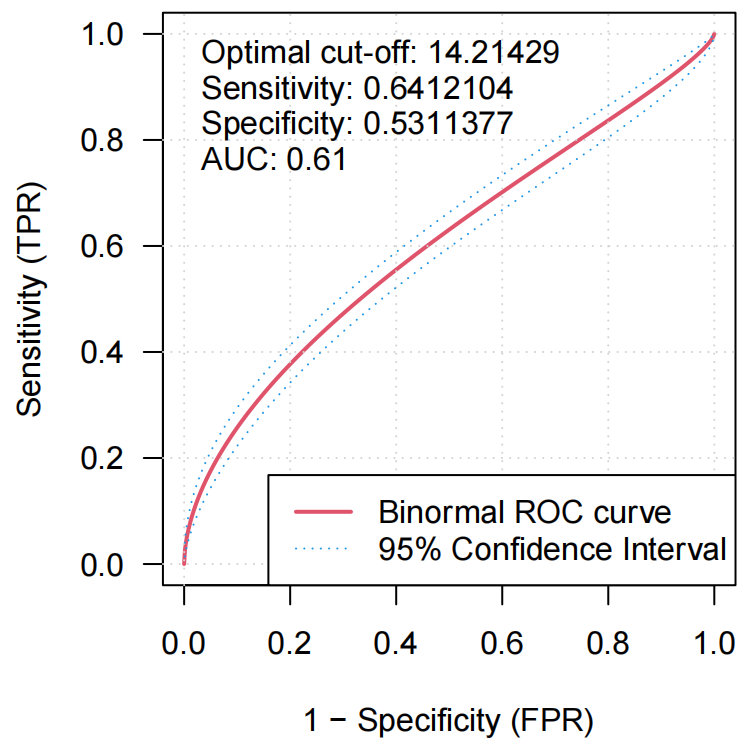

Supplement: Supplementary file 1 — Figures S1–S3. [file CAM4-14-e70527-s008.zip › cam470527-sup-0003-FigureS3.png]
