# Supplementary material for: The Association Between Neutrophil‐Percentage‐to‐Albumin Ratio (NPAR) and Mortality Among Individuals With Cancer: Insights From National Health and Nutrition Examination Survey
Source: Cancer Med. 2025 Jan 20;14(2):e70527. doi: 10.1002/cam4.70527 (PMC11744675; doi:10.1002/cam4.70527)
Supplement: Supplementary file 2 — Table S1. [file CAM4-14-e70527-s002.docx]

| Table S1. Baseline characteristics of study participants after propensity score matching in NHANES 2005–2016 | | | |
| --- | --- | --- | --- |
| Variables | Non-cancer,  N = 2,376 | Cancer,  N = 2,376 | *p* value |
| Age, years | 65.24 (14.07) | 65.33 (14.11) | 0.830 |
| Gender |  |  | 0.794 |
| Male | 1139 (47.9) | 1129 (47.5) |  |
| Female | 1237 (52.1) | 1247 (52.5) |  |
| Ethnicity |  |  | 0.975 |
| Non-Hispanic white | 1690 (71.1) | 1697 (71.4) |  |
| Non-Hispanic black | 318 (13.4) | 313 (13.2) |  |
| Mexican American | 150 (6.3) | 144 (6.1) |  |
| Other race | 218 (9.2) | 222 (9.3) |  |
| Education |  |  | 0.661 |
| Below high school level | 469 (19.7) | 490 (20.6) |  |
| High school | 522 (22.0) | 530 (22.3) |  |
| Above high school | 1385 (58.3) | 1356 (57.1) |  |
| Marital |  |  | 0.211 |
| Married/living with partner | 1495 (62.9) | 1446 (60.9) |  |
| Widowed/divorced/separated | 759 (31.9) | 786 (33.1) |  |
| Never married | 122 (5.1) | 144 (6.1) |  |
| Drinking |  |  | 0.480 |
| Never | 280 (11.8) | 306 (12.9) |  |
| Former | 582 (24.5) | 586 (24.7) |  |
| Current | 1514 (63.7) | 1484 (62.5) |  |
| Smoking |  |  | 0.970 |
| Never | 1065 (44.8) | 1057 (44.5) |  |
| Former | 940 (39.6) | 944 (39.7) |  |
| Current | 371 (15.6) | 375 (15.8) |  |
| BMI (kg/m2) | 28.78 (6.12) | 29.06 (6.50) | 0.130 |
| Poverty-to-income ratio |  |  | 0.805 |
| Poor (≤1) | 341 (14.4) | 348 (14.6) |  |
| Not poor (>1) | 2035 (85.6) | 2028 (85.4) |  |
| Hypertension |  |  | 0.002 |
| No | 963 (40.5) | 856 (36.0) |  |
| Yes | 1413 (59.5) | 1520 (64.0) |  |
| Hyperlipidemia |  |  | 0.060 |
| No | 457 (19.2) | 406 (17.1) |  |
| Yes | 1919 (80.8) | 1970 (82.9) |  |
| Diabetes |  |  | 0.014 |
| No | 1805 (76.0) | 1730 (72.8) |  |
| Yes | 571 (24.0) | 646 (27.2) |  |
| NPAR | 14.18 (2.72) | 14.38 (2.81) | 0.014 |
| Q1 | 473 (19.9) | 429 (18.1) |  |
| Q2 | 516 (21.7) | 497 (20.9) |  |
| Q3 | 634 (26.7) | 630 (26.5) |  |
| Q4 | 753 (31.7) | 820 (34.5) |  |
| Albumin, g/dL | 4.21 (0.32) | 4.19 (0.33) | 0.062 |
| Neutrophil percent, % | 59.23 (9.82) | 59.79 (10.06) | 0.053 |
| Neutrophil count, 10^9^/L | 4.28 (1.62) | 4.33 (1.67) | 0.254 |
| Lymphocyte count, 10^9^/L | 2.04 (1.55) | 2.13 (3.29) | 0.234 |
| Note: Continuous variables were expressed by the mean (SD), including Age, BMI, NPAR, Albumin, Neutrophil percent, Neutrophil count, Lymphocyte count. Categorical variables were expressed by the column percentage, including Gender, Ethnicity, Education, Marital, Drinking, Smoking, Poverty-to-income ratio, Hypertension, Hyperlipidemia, Diabetes, Q1, Q2, Q3, Q4. | | | |
