# Supplementary material for: The Association Between Neutrophil‐Percentage‐to‐Albumin Ratio (NPAR) and Mortality Among Individuals With Cancer: Insights From National Health and Nutrition Examination Survey
Source: Cancer Med. 2025 Jan 20;14(2):e70527. doi: 10.1002/cam4.70527 (PMC11744675; doi:10.1002/cam4.70527)
Supplement: Supplementary file 3 — Table S2. [file CAM4-14-e70527-s004.docx]

| Table S2. Analysis for the association between NPAR and risk of cancer | | | | |
| --- | --- | --- | --- | --- |
|  | Univariate analysis | | Multivariate analysis | |
| Variables | OR (95% CI) | *p* value | OR (95% CI) | *p* value |
| NPAR | 1.10 (1.07,1.12) | <0.0001 | 1.07 (0.84,1.36) | 0.59 |
| Q1 | ref | ref | ref |  |
| Q2 | 1.15 (0.97,1.36) | 0.11 | 0.97 (0.76,1.25) | 0.83 |
| Q3 | 1.47 (1.20,1.79) | <0.001 | 0.99 (0.69,1.41) | 0.96 |
| Q4 | 1.78 (1.49,2.13) | <0.0001 | 0.98 (0.60,1.59) | 0.92 |
| Albumin, g/dL | 0.50 (0.44,0.58) | <0.0001 | 1.07 (0.40,2.83) | 0.89 |
| Neutrophil percent, % | 1.02 (1.01,1.02) | <0.0001 | 0.99 (0.92,1.05) | 0.69 |
| Neutrophil count, 10^9^/L | 1.01 (0.97,1.04) | 0.65 | - | - |
| Lymphocyte count, 10^9^/L | 0.89 (0.76,1.04) | 0.15 | - | - |
| Age, years | 1.07 (1.06,1.07) | <0.0001 | 1.06 (1.06,1.07) | <0.0001 |
| Gender |  |  |  |  |
| Male | ref | ref | ref | ref |
| Female | 1.31 (1.18,1.46) | <0.0001 | 1.26 (1.11,1.43) | <0.001 |
| Ethnicity |  |  |  |  |
| Non-Hispanic white | ref | ref | ref | ref |
| Non-Hispanic black | 0.34 (0.30,0.39) | <0.0001 | 0.45 (0.39,0.53) | <0.0001 |
| Mexican American | 0.19 (0.15,0.25) | <0.0001 | 0.39 (0.31,0.50) | <0.0001 |
| Other race | 0.35 (0.27,0.44) | <0.0001 | 0.52 (0.41,0.66) | <0.0001 |
| Education |  |  |  |  |
| Below high school level | ref | ref | ref | ref |
| High school | 1.19 (0.98,1.44) | 0.07 | 1.18 (0.97,1.45) | 0.10 |
| Above high school | 1.44 (1.22,1.70) | <0.0001 | 1.76 (1.48,2.11) | <0.0001 |
| Marital |  |  |  |  |
| Married/living with partner | ref | ref | ref | ref |
| Widowed/divorced/separated | 1.59 (1.42,1.79) | <0.0001 | 0.96 (0.84,1.10) | 0.53 |
| Never married | 0.31 (0.24,0.39) | <0.0001 | 0.84 (0.65,1.10) | 0.20 |
| Drinking |  |  |  |  |
| Never | ref | ref | ref | ref |
| Former | 1.45 (1.19,1.77) | <0.001 | 1.14 (0.91,1.43) | 0.25 |
| Current | 1.01 (0.84,1.20) | 0.95 | 1.26 (1.03,1.53) | 0.02 |
| Smoking |  |  |  |  |
| Never | ref | ref | ref | ref |
| Former | 1.96 (1.75,2.20) | <0.0001 | 1.28 (1.12,1.46) | <0.001 |
| Current | 0.92 (0.78,1.08) | 0.31 | 1.36 (1.13,1.64) | 0.001 |
| BMI (kg/m2) | 1.00 (0.99,1.01) | 0.58 | - | - |
| Poverty-to-income ratio |  |  |  |  |
| Poor (≤1) | ref | ref | ref | ref |
| Not poor (>1) | 1.64 (1.37,1.97) | <0.0001 | 1.06 (0.94,1.20) | 0.88 |
| Hypertension |  |  |  |  |
| No | ref | ref | ref | ref |
| Yes | 2.46 (2.21,2.74) | <0.0001 | 1.16 (0.97,1.39) | 0.34 |
| Hyperlipidemia  No | ref | ref | ref | ref |
| Yes | 2.10 (1.78,2.47) | <0.0001 | 1.09 (0.96,1.24) | 0.11 |
| Diabetes |  |  |  |  |
| No | ref | ref | ref | ref |
| Yes | 1.88 (1.69,2.09) | <0.0001 | 0.88 (0.74,1.06) | 0.19 |
| Note: Adjusted for age, gender, ethnicity, education level, marital, drinking status, smoking status, Poverty-to-income ratio, hypertension, hyperlipidemia, diabetes, albumin, neutrophil percent.  Abbreviations: *OR*, odds ratio; *CI*, confidence interval; *BMI*, body mass index; *NPAR*, neutrophil percentage-to-albumin ratio. | | | | |
