# Supplementary material for: The Association Between Neutrophil‐Percentage‐to‐Albumin Ratio (NPAR) and Mortality Among Individuals With Cancer: Insights From National Health and Nutrition Examination Survey
Source: Cancer Med. 2025 Jan 20;14(2):e70527. doi: 10.1002/cam4.70527 (PMC11744675; doi:10.1002/cam4.70527)
Supplement: Supplementary file 4 — Table S3. [file CAM4-14-e70527-s005.docx]

| Table S3. Characteristics of the study cancer patients according to NPAR | | | | | | | |
| --- | --- | --- | --- | --- | --- | --- | --- |
| Variables | | Total, N=2,376 | Quartile 1,  N=594 | Quartile 2, N=592 | Quartile 3, N=598 | Quartile 4, N=592 | *p* value |
| Age, years | 62.46 (0.39) | | 60.11 (0.68) | 61.05 (0.63) | 63.44 (0.84) | 65.94 (0.76) | <0.0001 |
| Gender |  | |  |  |  |  | 0.57 |
| Male | 1129 (47.52) | | 269 (43.02) | 274 (43.54) | 287 (41.36) | 299 (46.27) |  |
| Female | 1247 (52.48) | | 325 (56.98) | 318 (56.46) | 311 (58.64) | 293 (53.73) |  |
| Ethnicity |  | |  |  |  |  | 0.03 |
| Non-Hispanic white | 1697 (71.42) | | 387 (85.34) | 435 (89.82) | 440 (87.82) | 435 (88.30) |  |
| Non-Hispanic black | 313 (13.17) | | 106 (6.39) | 67 (3.75) | 60 (3.36) | 80 (5.37) |  |
| Mexican American | 144 (6.06) | | 37 (2.12) | 32 (1.96) | 40 (2.33) | 35 (2.07) |  |
| Other race | 222 (9.34) | | 64 (6.15) | 58 (4.47) | 58 (6.49) | 42 (4.26) |  |
| Education |  | |  |  |  |  | 0.38 |
| Below high school level | 490 (20.62) | | 116 (12.34) | 114 (10.94) | 123 (11.95) | 137 (14.76) |  |
| High school | 530 (22.31) | | 127 (17.84) | 132 (21.39) | 145 (22.66) | 126 (20.96) |  |
| Above high school | 1356 (57.07) | | 351 (69.81) | 346 (67.67) | 330 (65.39) | 329 (64.29) |  |
| Marital |  | |  |  |  |  | 0.01 |
| Married/living with partner | 1446 (60.86) | | 386 (70.18) | 364 (67.54) | 362 (63.54) | 334 (60.40) |  |
| Widowed/divorced/separated | 786 (33.08) | | 162 (22.53) | 197 (27.21) | 196 (30.32) | 231 (35.28) |  |
| Never married | 144 (6.06) | | 46 (7.28) | 31 (5.25) | 40 (6.14) | 27 (4.32) |  |
| Drinking |  | |  |  |  |  | < 0.001 |
| Never | 306 (12.88) | | 78 (10.16) | 77 (9.87) | 68 (9.09) | 83 (12.01) |  |
| Former | 586 (24.66) | | 132 (17.09) | 126 (15.97) | 151 (19.97) | 177 (28.27) |  |
| Current | 1484 (62.46) | | 384 (72.74) | 389 (74.16) | 379 (70.94) | 332 (59.72) |  |
| Smoking |  | |  |  |  |  | 0.49 |
| Never | 1057 (44.49) | | 294 (49.33) | 264 (45.05) | 260 (45.91) | 239 (40.61) |  |
| Former | 944 (39.73) | | 207 (34.75) | 234 (38.74) | 254 (38.74) | 249 (42.23) |  |
| Current | 375 (15.78) | | 93 (15.91) | 94 (16.21) | 84 (15.35) | 104 (17.16) |  |
| BMI (kg/m2) | 28.87 (0.15) | | 27.75 (0.24) | 28.27 (0.31) | 29.46 (0.31) | 30.26 (0.41) | <0.0001 |
| Poverty-to-income ratio |  | |  |  |  |  | 0.31 |
| Poor (≤1) | 348 (14.65) | | 84 (8.30) | 89 (10.55) | 82 (8.10) | 93 (10.10) |  |
| Not poor (>1) | 2028 (85.35) | | 510 (91.70) | 503 (89.45) | 516 (91.90) | 499 (89.90) |  |
| Hypertension |  | |  |  |  |  | <0.0001 |
| No | 856 (36.03) | | 241 (49.26) | 232 (46.27) | 212 (40.79) | 171 (31.32) |  |
| Yes | 1520 (63.97) | | 353 (50.74) | 360 (53.73) | 386 (59.21) | 421 (68.68) |  |
| Hyperlipidemia |  | |  |  |  |  | 0.86 |
| No | 406 (17.09) | | 105 (17.66) | 92 (15.80) | 100 (16.82) | 109 (17.80) |  |
| Yes | 1970 (82.91) | | 489 (82.34) | 500 (84.20) | 498 (83.18) | 483 (82.20) |  |
| Diabetes |  | |  |  |  |  | <0.0001 |
| No | 1730 (72.81) | | 458 (82.59) | 446 (81.28) | 432 (76.89) | 394 (70.08) |  |
| Yes | 646 (27.19) | | 136 (17.41) | 146 (18.72) | 166 (23.11) | 198 (29.92) |  |
| NPAR | 14.21 (0.07) | | 11.11(0.08) | 13.51 (0.02) | 15.13 (0.03) | 17.74 (0.08) | <0.0001 |
| Albumin, g/dL | 4.23 (0.01) | | 4.37 (0.01) | 4.33 (0.01) | 4.19 (0.01) | 3.99 (0.02) | <0.0001 |
| Neutrophil percent, % | 59.71 (0.25) | | 48.56 (0.34) | 58.46 (0.18) | 63.34 (0.25) | 70.47 (0.26) | <0.0001 |
| Neutrophil count, 10^9^/L | 4.32 (0.05) | | 3.17 (0.05) | 3.98 (0.06) | 4.62 (0.08) | 5.77 (0.11) | <0.0001 |
| Lymphocyte count, 10^9^/L | 2.07 (0.03) | | 2.82 (0.13) | 2.00 (0.03) | 1.82 (0.03) | 1.55 (0.03) | <0.0001 |
| Abbreviations: *BMI*, body mass index; *NPAR*, neutrophil percentage-to-albumin ratio. | | | | | | | |
