# Supplementary material for: The Association Between Neutrophil‐Percentage‐to‐Albumin Ratio (NPAR) and Mortality Among Individuals With Cancer: Insights From National Health and Nutrition Examination Survey
Source: Cancer Med. 2025 Jan 20;14(2):e70527. doi: 10.1002/cam4.70527 (PMC11744675; doi:10.1002/cam4.70527)
Supplement: Supplementary file 5 — Table S4. [file CAM4-14-e70527-s001.docx]

| Table S4. Characteristics of the study prostate cancer patients according to NPAR | | | | | | | |
| --- | --- | --- | --- | --- | --- | --- | --- |
| Variables | | Total, N=104 | Quartile 1,  N=26 | Quartile 2, N=26 | Quartile 3, N=26 | Quartile 4, N=26 | *p* value |
| Age, years | 68.67(1.05) | | 65.39(1.58) | 69.75(1.47) | 69.19(2.89) | 71.28(2.12) | 0.14 |
| Ethnicity |  | |  |  |  |  | 0.5 |
| Non-Hispanic white | 63(60.58) | | 12(72.82) | 15(81.98) | 17(85.74) | 19(85.86) |  |
| Non-Hispanic black | 31(29.81) | | 12(16.95) | 10(15.52) | 6(9.63) | 3(5.43) |  |
| Mexican American | 4(3.85) | | 0(0.00) | 1(2.50) | 1(1.26) | 2(2.90) |  |
| Other race | 6(5.77) | | 2(10.23) | 0(0.00) | 2(3.38) | 2(5.81) |  |
| Education |  | |  |  |  |  | 0.09 |
| Below high school level | 27(25.96) | | 1(1.05) | 13(37.96) | 8(16.62) | 5(15.09) |  |
| High school | 18(17.31) | | 5(15.94) | 5(25.62) | 3(17.89) | 5(15.20) |  |
| Above high school | 59(56.73) | | 20(83.01) | 8(36.42) | 15(65.49) | 16(69.71) |  |
| Marital |  | |  |  |  |  | 0.93 |
| Married/living with partner | 82(78.85) | | 21(79.05) | 20(79.44) | 21(78.85) | 20(83.47) |  |
| Widowed/divorced/separated | 21(20.19) | | 5(20.95) | 5(18.95) | 5(21.15) | 6(16.53) |  |
| Never married | 1(0.96) | | 0(0.00) | 1(1.61) | 0(0.00) | 0(0.00) |  |
| Drinking |  | |  |  |  |  | 0.58 |
| Never | 7(6.73) | | 2(9.43) | 1(8.94) | 1(1.37) | 3(7.51) |  |
| Former | 28(26.92) | | 4(12.74) | 11(31.99) | 6(20.39) | 7(32.90) |  |
| Current | 69(66.35) | | 20(77.84) | 14(59.06) | 19(78.24) | 16(59.59) |  |
| Smoking |  | |  |  |  |  | 0.72 |
| Never | 40(38.46) | | 6(38.60) | 15(57.08) | 8(34.08) | 11(38.23) |  |
| Former | 56(53.85) | | 17(57.87) | 11(42.92) | 15(60.05) | 13(58.48) |  |
| Current | 8(7.69) | | 3(3.53) | 0(0.00) | 3(5.87) | 2(3.30) |  |
| BMI (kg/m2) | 28.93(0.74) | | 27.35(0.88) | 28.80(0.67) | 31.38(1.92) | 28.33(1.52) | 0.22 |
| Poverty-to-income ratio |  | |  |  |  |  | 0.14 |
| Poor (≤1) | 8(7.69) | | 0(0.00) | 4(9.87) | 4(5.93) | 0(0.00) |  |
| Not poor (>1) | 76(73.08) | | 26(100.00) | 22(90.13) | 22(94.07) | 26(100.00) |  |
| Hypertension |  | |  |  |  |  | 0.01 |
| No | 28(26.92) | | 10(52.75) | 3(10.79) | 10(49.83) | 5(12.55) |  |
| Yes | 1520 (63.97) | | 16(47.25) | 23(89.21) | 16(50.17) | 21(87.45) |  |
| Hyperlipidemia |  | |  |  |  |  | 0.2 |
| No | 16(15.38) | | 4(6.82) | 2(8.93) | 5(27.44) | 5(15.43) |  |
| Yes | 88(84.62) | | 22(93.18) | 24(91.07) | 21(72.56) | 21(84.57) |  |
| Diabetes |  | |  |  |  |  | 0.71 |
| No | 75(72.12) | | 22(81.18) | 14(66.44) | 17(75.76) | 22(81.36) |  |
| Yes | 29(27.88) | | 4(18.82) | 12(33.56) | 9(24.24) | 4(18.64) |  |
| Surgery |  | |  |  |  |  | 0.67 |
| No | 4(3.85) | | 1(2.54) | 0(0.00) | 2(4.14) | 1(2.93) |  |
| Yes | 100(96.15) | | 25(97.46) | 26(100.00) | 24(95.86) | 25(97.07) |  |
| Radiation |  | |  |  |  |  | 0.77 |
| No | 72(69.23) | | 21(70.43) | 17(66.39) | 18(76.00) | 16(60.01) |  |
| Yes | 32(30.77) | | 5(29.57) | 9(33.61) | 8(24.00) | 10(39.99) |  |
| Taking medicines |  | |  |  |  |  | 0.14 |
| No | 86(82.69) | | 22(72.51) | 20(81.72) | 23(94.61) | 21(76.06) |  |
| Yes | 18(17.31) | | 4(27.49) | 6(18.28) | 3( 5.39) | 5(23.94) |  |
| NPAR | 14.58(0.36) | | 11.46(0.64) | 13.92(0.05) | 15.40(0.12) | 18.26(0.42) | <0.0001 |
| Albumin, g/dL | 4.15(0.04) | | 4.23(0.04) | 4.35(0.05) | 4.13(0.05) | 3.90(0.10) | 0.01 |
| Neutrophil percent, % | 60.22(1.32) | | 48.77(2.82) | 60.51(0.91) | 63.55(0.82) | 70.86(0.94) | <0.0001 |
| Neutrophil count, 10^9^/L | 4.14(0.15) | | 3.13(0.20) | 4.15(0.25) | 4.32(0.27) | 5.23(0.35) | <0.001 |
| Lymphocyte count, 10^9^/L | 2.31(0.62) | | 4.06(2.00) | 1.78(0.07) | 1.59(0.11) | 1.34(0.11) | 0.04 |
| Abbreviations: *BMI*, body mass index; *NPAR*, neutrophil percentage-to-albumin ratio. | | | | | | | |
