# Supplementary material for: The Association Between Neutrophil‐Percentage‐to‐Albumin Ratio (NPAR) and Mortality Among Individuals With Cancer: Insights From National Health and Nutrition Examination Survey
Source: Cancer Med. 2025 Jan 20;14(2):e70527. doi: 10.1002/cam4.70527 (PMC11744675; doi:10.1002/cam4.70527)
Supplement: Supplementary file 6 — Table S5. [file CAM4-14-e70527-s006.docx]

| Table S5. The relationship between NPAR and all-cause mortality among prostate cancer patients in NHANES 2005–2010 | | | | |
| --- | --- | --- | --- | --- |
| Cancer | Character | Range median (IQR) | aHR ^a^ (95% CI) | *p* value |
| Prostate cancer | NPAR |  | 1.22(1.03, 1.44) | 0.02 |
| (n=104) | Q1 | 12.07(2.62-12.97) | ref | ref |
|  | Q2 | 14.04(12.97-14.57) | 6.07(1.52,24.24) | 0.01 |
|  | Q3 | 15.38(14.57-16.20) | 1.55(0.22,11.12) | 0.66 |
|  | Q4 | 18.19(16.20-20.81) | 8.71(1.43,53.24) | 0.02 |
|  | P for trend | |  | 0.076 |
| Note: Cox proportional hazards regression for the relationship between NPAR and mortality among cancer patients. aHR ^a^: Adjusted for age, gender, ethnicity, education level, marital, drinking status, smoking status, Poverty-to-income ratio, surgery, radiotherapy, medicine, hypertension, hyperlipidemia and diabetes.  Abbreviations: *HR*, hazard ratio; *CI*, confidence interval; *IQR*, Interquartile range; *NPAR*, neutrophil percentage-to-albumin ratio. | | | | |
