# Supplementary material for: The Association Between Neutrophil‐Percentage‐to‐Albumin Ratio (NPAR) and Mortality Among Individuals With Cancer: Insights From National Health and Nutrition Examination Survey
Source: Cancer Med. 2025 Jan 20;14(2):e70527. doi: 10.1002/cam4.70527 (PMC11744675; doi:10.1002/cam4.70527)
Supplement: Supplementary file 7 — Table S6. [file CAM4-14-e70527-s007.docx]

| Table S6. Post hoc test analysis of NPAR quartiles in cancer-related deaths. | | | | | |
| --- | --- | --- | --- | --- | --- |
| Tukey's multiple comparisons test | Mean Diff. | 95.00% CI of diff. | Below threshold? | Summary | Adjusted P Value |
| Q1 vs. Q2 | -3.148 | -4.005 to -2.291 | Yes | **** | <0.0001 |
| Q1 vs. Q3 | -5.016 | -5.873 to -4.159 | Yes | **** | <0.0001 |
| Q1 vs. Q4 | -8.359 | -9.215 to -7.502 | Yes | **** | <0.0001 |
| Q2 vs. Q3 | -1.868 | -2.725 to -1.011 | Yes | **** | <0.0001 |
| Q2 vs. Q4 | -5.21 | -6.067 to -4.354 | Yes | **** | <0.0001 |
| Q3 vs. Q4 | -3.343 | -4.200 to -2.486 | Yes | **** | <0.0001 |

Note: The NPAR data came from 224 tumor patients who died from cancer. Tukey's multiple comparisons test was used as a post-hoc test.
