# Supplementary material for: The Association Between Neutrophil‐Percentage‐to‐Albumin Ratio (NPAR) and Mortality Among Individuals With Cancer: Insights From National Health and Nutrition Examination Survey
Source: Cancer Med. 2025 Jan 20;14(2):e70527. doi: 10.1002/cam4.70527 (PMC11744675; doi:10.1002/cam4.70527)
Supplement: Supplementary file 8 — Table S7. [file CAM4-14-e70527-s003.docx]

| Table S7. Analysis of log-rank test for survival prognosis in cancer-related deaths across different NPAR quartiles. | | | | |
| --- | --- | --- | --- | --- |
| Log-rank test | Hazard Ratio | 95% CI of ratio | Summary | P Value |
| Q1 vs. Q2 | 0.9671 | 0.6677 to 1.401 | ns | 0.8568 |
| Q1 vs. Q3 | 1.021 | 0.7047 to 1.478 | ns | 0.9122 |
| Q1 vs. Q4 | 0.6335 | 0.4332 to 0.9265 | * | 0.0122 |
| Q2 vs. Q3 | 1.040 | 0.7181 to 1.506 | ns | 0.8319 |
| Q2 vs. Q4 | 0.6637 | 0.4547 to 0.9688 | * | 0.0257 |
| Q3 vs. Q4 | 0.6197 | 0.4234 to 0.9072 | ** | 0.0089 |

Note: The survival data came from 224 tumor patients who died from cancer. Log-rank test was used to analyze the differences of survival prognosis in quartiles.
